# Asexual reproduction changes predator population dynamics in a life predator–prey system

**DOI:** 10.1002/1438-390X.1017

**Published:** 2019-01-11

**Authors:** Thomas Scheuerl, Claus‐Peter Stelzer

**Affiliations:** ^1^ Research Institute for Limnology, University of Innsbruck Mondsee Austria

**Keywords:** *Brachionus calyciflorus*, population dynamics, sex

## Abstract

Many organisms display oscillations in population size. Theory predicts that these fluctuations can be generated by predator–prey interactions, and empirical studies using life model systems, such as a rotifer‐algae community consisting of *Brachionus calyciflorus* as predator and *Chlorella vulgaris* as prey, have been successfully used for studying such dynamics. *B. calyciflorus* is a cyclical parthenogen (CP) and clones often differ in their sexual propensity, that is, the degree to which they engage into sexual or asexual (clonal) reproduction. Since sexual propensities can affect growth rates and population sizes, we hypothesized that this might also affect population oscillations. Here, we studied the dynamical behaviour of *B. calyciflorus* clones representing either CPs (regularly inducing sex) or obligate parthenogens (OPs). We found that the amplitudes of population cycles to be increased in OPs at low nutrient levels. Several other population dynamic parameters seemed unaffected. This suggests that reproductive mode might be an important additional variable to be considered in future studies of population oscillations.

## Graphical abstract

Many organisms find themselves in predator‐prey relationships where growth rates of prey and attack rates of predators determine the dynamics. Since sexual reproduction can affect growth rates and population sizes compared to asexual reproduction, we hypothesized that this might also affect population oscillations. Using a predator‐prey system with *Brachionus calyciflorus* rotifer clones representing either cyclical parthenogens (regularly inducing sex) or obligate parthenogens, feeding on *Chlorella vulgaris* algal populations, we found that the amplitudes of predator population cycles are increased in obligate parthenogens at low nutrient levels.

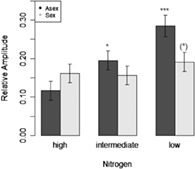

## 1 INTRODUCTION

Population oscillations have been shown in many natural animal populations (e.g., Fussmann, Ellner, Shertzer, & Hairston, 2000; Stenseth, Falck, Bjørnstad, & Krebs, 1997; Yoshida, Jones, Ellner, Fussmann, & Hairston, 2003). While populations may simply fluctuate around steady‐state or equilibrium densities, interactions in communities can lead to population fluctuations such as limit cycle patterns (May, 1972). Classical population interactions that lead to population oscillations are predator–prey communities (Fussmann et al., 2000; Stenseth et al., 1997). The resulting predator–prey cycles have been mathematically predicted by theoretical models (May, 1972; Rosenzweig, 1971; Volterra, 1926) and verified in many experiments (e.g., Fussmann et al., 2000; Yoshida et al., 2003). Further, such cycles can be affected by nutrient enrichment, which might affect population stability (Rosenzweig, 1971), potentially resulting in chaotic dynamics with increased extinction risk. Experimental model systems, such as the rotifer *Brachionus calyciflorus* and its prey alga *Chlorella vulgaris* have been successfully used to increase our mechanistic understanding of population cycles (Fussmann et al., 2000; Yoshida et al., 2003). In this system, different nitrogen levels can result in different population dynamics (Fussmann et al., 2000). In all mathematical models describing population cycles, the population growth rate *r* and the carrying capacity *K* are critical parameters (Fussmann et al., 2000; May, 1972; Rosenzweig, 1971; Volterra, 1926) determining the limit cycle pattern, which was also shown for this rotifer‐algae system (Fussmann et al., 2000). Because *B. calyciflorus* is a cyclical parthenogen (CP), *r* and *K* are intrinsically connected to the ratio of sexual and asexual reproduction, and both parameters may display great variation among clones (Scheuerl & Stelzer, 2013, 2017; Stelzer, Schmidt, Wiedlroither, & Riss, 2010). Obligate parthenogens typically grow much quicker and reach higher population densities compared with CPs (Scheuerl, Riss, & Stelzer, 2011).

*B. calyciflorus* females normally reproduce by ameiotic parthenogenesis (Supporting Information Figure S1), but they can also initiate sporadic sexual episodes (Nogrady, Wallace, & Snell, 1993) with haploid dwarf males (Fussmann, 2011). Induction of sexuality is density dependent (Gilbert, 2003; Schröder, 2005; Stelzer & Snell, 2003), differs among clones (Scheuerl et al., 2011) and chemical cues induce the production of sexual females (Snell et al., 2006; Snell & Stelzer, 2005; Stelzer & Snell, 2003; Timmermeyer & Stelzer, 2006). The oocytes of these sexual females undergo meiosis and develop into males (if not fertilized) or diploid diapausing eggs (if fertilized). In previous studies, we have identified rotifer clones in which sexual reproduction was completely lost (obligate parthenogens [OPs]). This loss of sexual reproduction is controlled by a single locus (Scheuerl et al., 2011; Stelzer et al., 2010). Clones homozygous for a mutant allele (genotype: *op/op*) are OPs while heterozygous clones (+/*op*), or clones homozygous for the wild‐type allele (+/+) are CPs. All three genotypes can be experimentally generated by intercrossing or self‐fertilization of heterozygous clones (Scheuerl et al., 2011; Stelzer et al., 2010).

Since in cyclical parthenogenetic clones male production affects the maximum growth rate and the maximum population density (Scheuerl et al., 2011), we hypothesized that OPs (not producing males) should exhibit different predator–prey cycles compared with CPs. Obligate asexual clones should grow faster and reach higher densities, since population size is not regulated by sexual induction, and this might ultimately change the population oscillation pattern compared with sexual clones. This hypothesis has not been addressed before—all previous work in these OP clones has been done under experimental conditions where rotifers and algae could not directly interact because food algae were grown in a separate container and fed to the rotifer populations at a constant rate (Stelzer, 2002).

Here, we compared population dynamics of cyclical and obligatory parthenogenetic monogonont rotifers (*B. calyciflorus*), while predating on *C. vulgaris* algae in the same environment. Using a two‐factor crossed design, with three nitrogen levels and two reproductive modes (CP, OP), we found that oscillations of OPs were characterized by higher amplitudes, in particular when nitrogen concentrations are low. These findings were compared with theoretical predictions from previously published mathematical models.

## 2 MATERIALS AND METHODS

### 2.1 Origin of clones and general culture conditions

We used cyclical parthenogenetic clones, which were all homozygous for the wild‐type allele for sexual reproduction (genotype: +/+) and obligate parthenogenetic clones homozygous for the mutant allele (genotype: *op*/*op*). All clones were established by self‐fertilization from one heterozygous clone (+/*op*) and thus shared the same level of homozygosity (at other genes than the *op* locus) and experienced the same amount of inbreeding (+/*op*, clone “Florida 23” in Scheuerl et al. (2011)). Thus, all experimental clones derived from one stem female, but they harbored a small amount of genetic variation since they were all produced by sexual reproduction.

Stem cultures of rotifers and algae were cultured in COMBO medium (Kilham, Kreeger, Lynn, Goulden, & Herrera, 1998) and maintained at 21°C. Continuous illumination was provided with daylight fluorescent bulbs (30–40 μE m^−2^ s^−1^ for rotifers; 200 μE m^−2^ s^−1^ for algae *C. vulgaris*).

### 2.2 Chemostat experiment

We followed the population dynamics of our predator–prey system in single‐stage chemostats (Stelzer, 2009). We used a monoclonal culture of *C. vulgaris*, which we obtained after repeated plating on COMBO agar. For the experiment, we used six treatments, with the two reproductive modes and three different concentrations of nitrogen (60, 120 and 240 μM). We used four replicates per treatment, which resulted in a total of 24 chemostats. Since rotifers were previously fed with *Chlamydomonas reinhardtii*, the remaining algae in rotifer cultures were killed in a 0.0001% Polyhexamethylenbiguanid, a substance which is commonly used in commercial pool cleaners (Revacil®).

The population dynamics of predators were monitored using an automated sampling system (Stelzer, 2009). Briefly, this computer‐controlled system sampled our 24 chemostat populations and took digital images of the cultures at 6‐hr intervals (53 days with a total of 212 observations). The digital images were analyzed by image analysis algorithms that allowed estimation of females and male density at a precision comparable with conventional counting techniques (Stelzer, 2009). Individual abundance estimates were based on ~200 individuals per sampling event. Algal concentrations were estimated from absorbance readings at 470 nm wavelength using a photometer (Vernier®‐colorimeter, model COL‐BTA) with a flow‐through cuvette. In rare cases, observed algal wall growth was removed using a magnetic stir bar inside the chemostat. Chemostats were continuously aerated and illuminated at 120 μE m^−2^ s^−1^. Fresh medium was provided from 20 L bottles at a dilution rate of 0.6 d^−1^ for all chemostats; containing 380 mL volume.

### 2.3 Raw data preparation

All calculations were done with the NLTSM program provided in Turchin (2003). NLTSM encompasses a phenomenological characterization of each time series and yields a number of response variables, such as: (a) the amplitude *S* of population fluctuations (measured as the *SD* of log‐transformed population densities), (b) the period length *T*, where the autocorrelation function reaches its first maximum and (c), the Lyapunov exponent for each time series.

Our raw response variable was the time series of biovolume (μL/mL) of female rotifers. We used this measure instead of abundances (females per mL) because there are known differences in body size among clones with different reproductive modes (Scheuerl et al., 2011; Stelzer et al., 2010). The first 4 days of each time series were truncated since they contained the initial phase of population establishment. Time series data were quadratically detrended prior to NLTSM analysis. The parameter “base lag” in NLTSM program was set to five sampling intervals, which corresponds to a time period of 1.25 days in our 6 hr‐sampling schedule.

### 2.4 Statistical analysis

We used analysis of variance (ANOVA) in which response variables, amplitude, period length, Lyapunov exponent and coefficient of variance (extracted from the NLTSM analysis) where tested against factor levels of reproduction mode and nitrogen levels as fixed effects. All statistical analysis was done in R (R Development Core Team, 2010). Models were tested for simplification by dropping the interaction term, and in case significant interactions were retained, we used a posteriori Tukey tests to investigate pairwise differences between fixed effect factor levels.

### 2.5 Mathematical model

We used previously published mathematical models to obtain theoretical predictions about the *B. calyciflorus*—*C. vulgaris* population cycles under various nitrogen levels and reproduction modes. Fussmann et al. (2000) formulated such a model for predicting the dynamics between these two species under varying nitrogen levels. We combined this model with another model, describing how *B. calyciflorus* grows under various sex induction ratios (Fussmann, Ellner, & Hairston Jr, 2003). The model parameter was used as published in these two previous studies (Fussmann et al., 2000, 2003).

## 3 RESULTS

**Figure 1:**
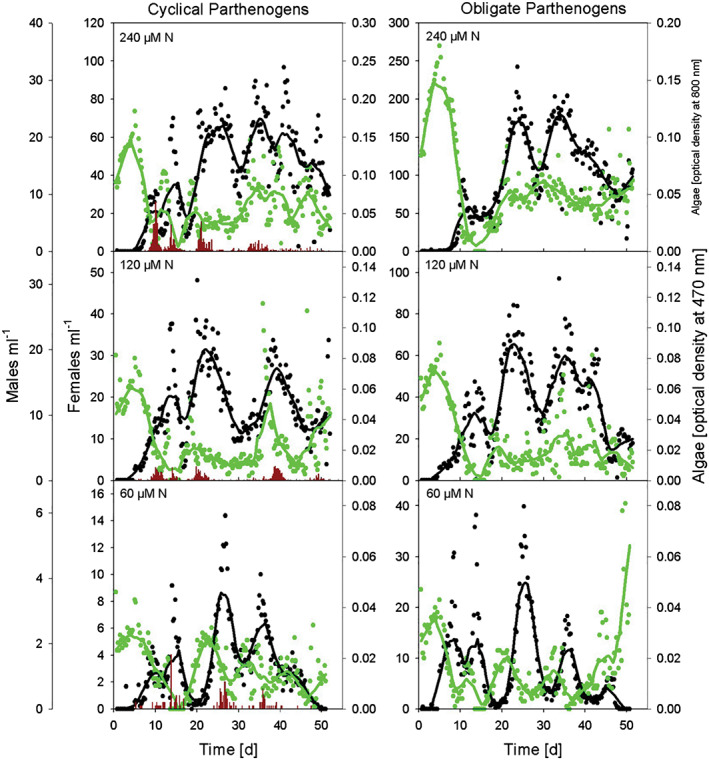
Population dynamics of cyclical versus obligate parthenogens at three different nutrient levels. One chemostat per treatment is presented to illustrate the interaction between the predator rotifer females and the prey algae of each treatment. Smoothed data of all other rotifer densities are presented in Figure S1. All populations showed oscillating population dynamics. Please note the different scales of the y axes. Lines were fitted with a negative exponential smoother with a sampling period of 0.1 (i.e., 20 observations or 5 days), third polynomial degree and rejected outliers. Black: density of rotifer females, green: algae. Small blue bars at the bottom indicate the density of males in cyclical parthenogens. Please note the different scales of the associated plot axes

All experimental communities of rotifers and algae displayed visual predator–prey cycles (Figure 1). The CPs regularly produced males. The raw data with rotifer female numbers indicate a higher maximum density (amplitudes) for OPs compared to CPs (Figure S2), which was also the case for biovolume. These higher amplitudes seem consistent across the three different nutrient levels. Independent from the nutrient level, all CPs produced males. However, the male numbers seemed to decrease with lower nitrogen levels. Algae populations also displayed some cyclic patterns depending on nitrogen level. Unfortunately, algae data from day 14 to day 16 are missing for most replicates; due to a technical failure of the photometer we used measuring algae density. Because of that, we excluded the algae data from further analysis.

**Figure 2:**
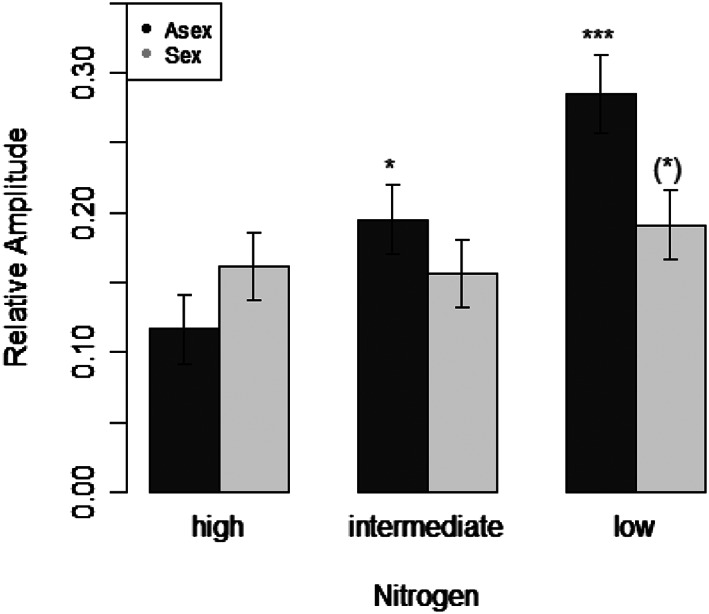
The relative amplitude for rotifer populations under three different nitrogen concentrations comparing obligate and cyclical parthenogens. Bars indicate the relative amplitude for low (60 μM), medium (120 μM) and high (240 μM) nitrogen levels (±*SE*, *n* = 4). Dark gray represents obligate parthenogens, while light gray represents cyclical parthenogens

From the time series of biovolume of female rotifers, we extracted various parameters describing oscillatory behavior using the NLTSM program (Turchin, 2003). From these parameters, only relative amplitude, thus height of the oscillations, seemed to be affected by the reproduction mode across nutrient levels. The relative amplitude *S* increased with decreasing nitrogen level for the OPs, while it was rather stable for the CPs (Figure 2). For the relative amplitude we found a significant interaction between reproduction mode and nitrogen level, indicating that CPs and OPs indeed displayed different oscillation patterns across nutrient levels (Table 1). A post hoc pairwise comparison test revealed differences between OPs at the 60 μM nitrogen level and the two CPs populations at 120 and 240 μM nitrogen levels (Table S1, Tukey test, *p*_adj_ value = 0.0326 and *p*_adj_ value = 0.0423, respectively). We also detected differences within the OPs between the 60 and 240 μM nitrogen treatment (Tukey test, *p* value = 0.0037). For the coefficient of variance, we obtained results similar to relative amplitude (Figure S3). As both parameters are tightly connected, this is not surprising. Again, we found a significant interaction between reproduction mode and nitrogen level (ANOVA, *F* = 5.54, *df* = 2.17, *p* value = 0.014). In the following Tukey test several pairwise comparisons were significant (Table S2). Because the coefficient of variance is not a parameter obtained in the NLTSM analysis and is highly similar to the amplitude, we did not consider it in further detail. In any case, these findings support the impression that oscillations change toward higher fluctuations around the mean.

**Table 1:** Analysis of variance showed significant interactions between reproduction mode and nitrogen level

	*df*	Sum square	Mean square	*F* value	*p* value
Nitrogen	2	0.03198	0.015991	6.63	0.00744**
Reproduction mode	1	0.00379	0.003785	1.569	0.22726
Nitrogen: Reproduction mode	2	0.01831	0.009157	3.797	0.04334*
Residuals	17	0.041	0.002412		

*** *p* = 0.001;

* *p* = 0.01.

**Figure 3:**
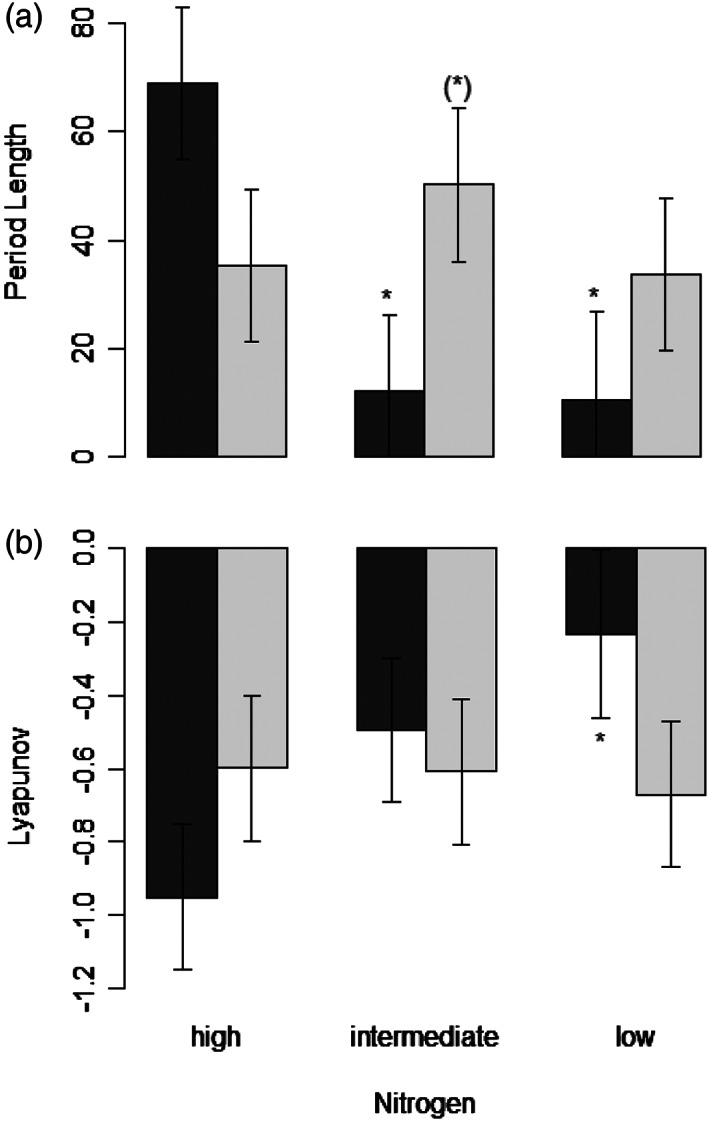
Period length (a) and Lyapunov exponent (b) from rotifer populations. Similar as in Figure 2, bars indicate parameters for low, medium and high nitrogen levels (±*SE*, *n* = 4). Dark gray represents obligate parthenogens (OPs), while light gray represents cyclical parthenogens (CPs)

The other parameters describing the population oscillations were not affected by reproduction mode or nitrogen level (Figure 3). There was a trend in period length to be shorter (i.e., higher oscillation frequency) with decreasing nitrogen level for OPs, while period length for CPs was clearly not affected (Figure 3a). However, this trend was not significant. Similarly, for the Lyapunov exponent, a measurement for chaos in time series (Rosenstein, Collins, De Luca, & Michael, 1993), we detected no significant effects, neither for reproduction mode nor nitrogen level. Lyapunov exponents were overall negative, thus no chaos pattern was detected. There was a nonsignificant trend in the Lyapunov exponent to turn from very negative to neutral with decreasing nitrogen level in OPs, while this was not the case in CPs (Figure 3b).

The theoretical model published by Fussmann et al. (2000) predicts profound changes in the population oscillations (Figure S4) along a gradient of nitrogen concentrations. The amplitude of an OP population is predicted to be about twice as high as for CPs (assuming the sex induction ratio published elsewhere; Fussmann et al., 2003, but similar to our clones; Scheuerl et al., 2011). Amplitudes increased at higher nitrogen levels, while at low nitrogen levels equilibria developed. The model further indicated an increased period length of the fluctuations for OPs, and stable population equilibria at low nitrogen levels.

## 4 DISCUSSION

Our results indicate that asexual populations display slight but significantly different population dynamic patterns. Under pure asexual reproduction, population sizes had a greater variance around the mean, indicated by higher amplitudes and higher coefficients of variance. Previous studies found that OPs have higher growth rates and population densities compared to CPs (Scheuerl et al., 2011). With populations growing into higher densities, it seems plausible that amplitudes of the oscillation increase. The final population size is also determined by nutrient level. Increased densities are expected at high nutrient levels, and we confirmed this pattern in our populations (Figure 1). Since algal growth is facilitated by higher nitrogen concentrations (Fussmann et al., 2000), rotifers should have more food available and thus reach higher densities. Both, the reproduction mode and the nitrogen level affected our rotifer populations. Interestingly, the highest relative amplitudes and greatest difference between sexual and asexual rotifers were observed at the lowest nitrogen level. This is contrary to predictions based on the “paradox of enrichment” hypothesis (Gilpin, 1972; May, 1972; Rosenzweig, 1971), which states that population fluctuations should increase at higher nutrient levels, and consequently stability should decrease (Fussmann et al., 2000). In contrast, we found our populations to fluctuate more strongly at low nutrient levels, potentially because the nitrogen‐dilution rate we used is predicted to be at the edge between stable equilibria and cycles (Fussmann et al., 2000). This might have resulted in cycles due to stochastic events. For OPs, our result matches a finding by other authors (McCauley, Nisbet, Murdoch, de Roos, & Gurney, 1999) who investigated population dynamics of *Daphnia*‐algae systems and observed destabilization after replacement of sexual with asexual stages in *Daphnia.* Here, resting eggs production stabilized the fluctuations by uncoupling high consumer dynamics from temporarily scarce food supply. In our system, the same energy channelling might have produced similar effects for CPs.

With increased amplitudes, one might also expect the periods of oscillations to change. If populations of predators grow into higher densities and decrease the prey population to a greater degree, recovery of the prey might take longer and thus result in increased oscillation periods. Our data suggest that this scenario is not likely. Period length and oscillation frequency were not affected by the reproduction mode. This might be explained by the time shift at which predators “follow” their prey. OPs will continue producing clonal offspring until algae reach very low concentrations. While this final offspring production would result in the increased population size of OPs, it might not affect prey populations anymore, because algal concentrations have declined to levels where encounters between predator and prey become unlikely. Under this scenario, the period length would not change, but this hypothesis needs to be checked by future studies.

Compared with the models of Fussmann et al. (2000, 2003), our experimental data show some similarities, but also differences. In agreement with the theoretical predictions, the amplitudes for OPs were increased, which supports the view that sexual reproduction limits the maximum population densities. Even though we directly used model parameters from the original publications instead of newly establishing such data for our clones, OP densities were predicted to be about twice as high as for CPs, which fits well to our observations (Figure S4). The model also predicted that different nitrogen levels alter the dynamics. However, the theoretical model predicted higher amplitudes with higher nitrogen levels while we observed the opposite. While the model predicted very low rotifer densities at the lows of the population cycle, our rotifer populations did not crash to such an extent but were maintained at some higher level (at 240 μM nitrogen we observed ~50 females per mL at the minimum, while the model predicted densities of 1–2 females per mL). This might explain the different effect in maximum amplitude. Further, the model suggested an effect on the period length such that OPs need more time to recover from a population crash, whereas our results only indicated a nonsignificant effect on period length. Finally, the model, as well as the experimental findings of Fussmann et al. (2000) indicated a higher destabilization at higher nitrogen levels, while we found our populations to be more stable at higher nitrogen levels. One could argue that wall growth, which is common in chemostat cultures, might have been an additional food source for the rotifers during through‐phases and that this effect might have been more pronounced in the high nitrogen treatments. This could serve as an additional explanation why rotifer populations usually never declined to low numbers as predicted by the model. However, we did not observe noticeable algal wall growth, and also bacterial wall growth seemed to be very low until the late stage of the experiment (probably due to the Polyhexamethylenbiguanid‐treatment of the rotifer eggs, and because we started with a monoclonal algae culture, which did not result in algal clumping evolution). Thus, we assume that wall growth did not have a strong influence on population dynamics, at least not during most of the duration of these experiments.

Finally, an intriguing idea would be that asexual reproduction comes with an ecological cost of population destabilization due to resource overexploitation. Sexual reproduction is predicted to increase adaptation of populations (Gray & Goddard, 2012; Kaltz & Bell, 2002; Scheuerl & Stelzer, 2013, 2017) and is thus important for the evolution of organisms (Bell, 1982; Maynard Smith, 1978). However, asexual reproduction has several theoretical advantages (Bell, 1982; Maynard Smith, 1978; Stelzer, 2015), for example, faster population growth. Asexual clones should quickly displace sexual populations within ecological times, that is, before evolutionary constrains can act (Bell, 1982; Maynard Smith, 1978). Resource overexploitation of asexually reproducing populations and a greater destabilization of such populations might be a new and interesting factor for explaining the long‐term persistence of sexual reproduction by a group‐selection mechanism (Nunney, 1989). Under this scenario, asexual populations might become established in some populations, but this population will go extinct due to their inherent instability. Our experiment did not directly confirm this prediction, but the increased relative amplitudes, which are often considered as an indicator of instability (McCauley et al., 1999), point somewhat into this direction. Mathematically stable equilibria show negative Lyapunov exponents, while chaotic dynamics would display positive exponents (Becks, Hilker, Malchow, Jürgens, & Arndt, 2005). We observed a (nonsignificant) trend in the Lyapunov exponent to move closer toward positive values in OPs (Figure 3b). Extrapolating this trend would suggest that chaotic dynamics might occur at nitrogen levels lower than those used in our experiment, which could be tested in future studies. Such future experiments would ideally involve more than four replicate populations (such as our study), and longer experimental runs, in order readdress the hypothesis that asexual populations are more prone to extinction by destabilized population dynamics.

## Supporting information


**Figure S1** *Brachionus calyciflorus* life cycle. Redrawn from Stelzer PNAS 2015 (Figure 2)
**Figure S2.** Population dynamics of cyclical versus obligate parthenogens at three different nutrient levels. Smoothed data for all chemostats are shown. All of the rotifer populations show oscillations. Lines were fitted with a negative exponential smoother with a sampling period of 0.1 (i.e., 20 observations or 5 days), third polynomial degree and rejected outliers
**Figure S3.** The coefficient of variance for rotifer populations under three different nitrogen concentrations comparing obligate and cyclical parthenogens. Bars indicate the relative amplitude for low (60 μM), medium (120 μM) and high (240 μM) nitrogen levels (±*SE*, *n* = 4). Dark gray represents obligate parthenogens, while light gray represents cyclical parthenogens
**Figure S4.** Theoretical predictions extracted from published models of the same experimental system. The model parameters were applied as presented by two models previously published to describe the dynamics between rotifers and algae under different nitrogen levels and different reproduction modes. Please note the different axis scales for each plot
**Table S1.** Tukey test on relative amplitude with reproduction mode (s = sex; a = asex) and nitrogen level as fixed effects
**Table S2.** Tukey test on coefficient of variance with reproduction mode and nitrogen level as fixed effects
