# Supplementary material for: Asexual reproduction changes predator population dynamics in a life predator–prey system
Source: Popul Ecol. 2019 Jan 11;61(2):210–6. doi: 10.1002/1438-390X.1017 (PMC7594307; doi:10.1002/1438-390X.1017)
Supplement: Supplementary file 1 — Figure S1 Brachionus calyciflorus life cycle. Redrawn from Stelzer PNAS 2015 (Figure 2) Figure S2. Population dynamics of cyclical versus obligate parthenogens at three different nutrient levels. Smoothed data for all chemostats are shown. All of the rotifer populations show oscillations. Lines were fitted with a negative exponential smoother with a sampling period of 0.1 (i.e., 20 observations or 5 days), third polynomial degree and rejected outliers Figure S3. The coefficient of variance for rotifer populations under three different nitrogen concentrations comparing obligate and cyclical parthenogens. Bars indicate the relative amplitude for low (60 μM), medium (120 μM) and high (240 μM) nitrogen levels (±SE, n = 4). Dark gray represents obligate parthenogens, while light gray represents cyclical parthenogens Figure S4. Theoretical predictions extracted from published models of the same experimental system. The model parameters were applied as presented by two models previously published to describe the dynamics between rotifers and algae under different nitrogen levels and different reproduction modes. Please note the different axis scales for each plot Table S1. Tukey test on relative amplitude with reproduction mode (s = sex; a = asex) and nitrogen level as fixed effects Table S2. Tukey test on coefficient of variance with reproduction mode and nitrogen level as fixed effects [file POPE-61-210-s001.doc]

Supplemental Material for

**Asexual reproduction changes predator population dynamics in a life predator-prey system**

**Thomas Scheuerl^*^ and Claus-Peter Stelzer^1^**

**Fig. S1.** ***Brachionus calyciflorus* life cycle. Redrawn from Stelzer PNAS 2015 (Fig.2).**


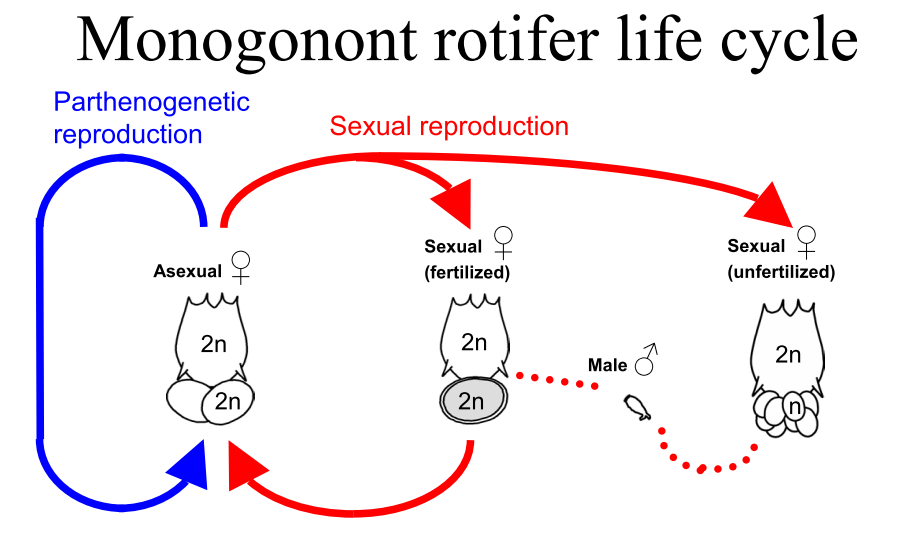


**Fig. S2. Population dynamics of cyclical vs. obligate parthenogens at three different nutrient levels.** Smoothed data for all chemostats are shown. All of the rotifer populations show oscillations. Lines were fitted with a negative exponential smoother with a sampling period of 0.1 (i.e. 20 observations, or 5 days), third polynomial degree and rejected outliers.

**Fig. S3. The coefficient of variance for rotifer populations under three different nitrogen concentrations comparing obligate and cyclical parthenogens.** Bars indicate the relative amplitude for low (60 µM), medium (120 µM) and high (240 µM) nitrogen levels (+ SE, n=4). Dark grey represents obligate parthenogens, while light grey represents cyclical parthenogens.


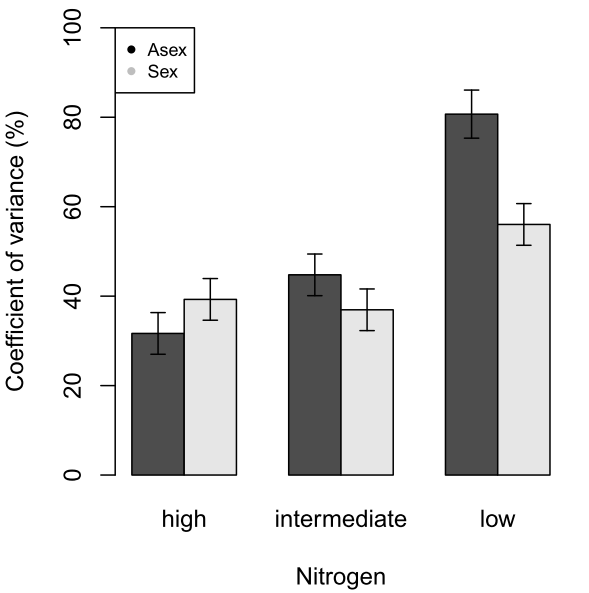


**Fig. S4. Theoretical predictions extracted from published models of the same experimental system.** The model parameters were applied as presented by two models previously published to describe the dynamics between rotifers and algae under different nitrogen levels and different reproduction modes. Please note the different axis scales for each plot.


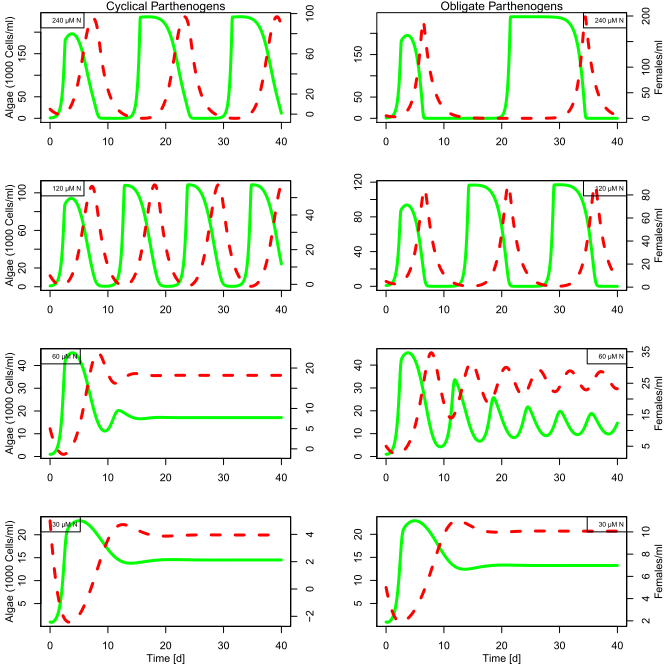


**Table S1.** Tukey test on relative amplitude with reproduction mode (s=sex-a=asex) and nitrogen level as fixed effects.

**Table S2.** Tukey test on coefficient of variance with reproduction mode and nitrogen level as fixed effects.
